# Tic62: a protein family from metabolism to protein translocation

**DOI:** 10.1186/1471-2148-7-43

**Published:** 2007-03-20

**Authors:** Mónica Balsera, Anna Stengel, Jürgen Soll, Bettina Bölter

**Affiliations:** 1 Dep Biologie I, Botanisches Institut, LMU München, 80638 München, Germany

## Abstract

**Background:**

The function and structure of protein translocons at the outer and inner envelope membrane of chloroplasts (Toc and Tic complexes, respectively) are a subject of intensive research. One of the proteins that have been ascribed to the Tic complex is Tic62. This protein was proposed as a redox sensor protein and may possibly act as a regulator during the translocation process. Tic62 is a bimodular protein that comprises an N-terminal module, responsible for binding to pyridine nucleotides, and a C-terminal module which serves as a docking site for ferredoxin-NAD(P)-oxido-reductase (FNR). This work focuses on evolutionary analysis of the Tic62-NAD(P)-related protein family, derived from the comparison of all available sequences, and discusses the structure of Tic62.

**Results:**

Whereas the N-terminal module of Tic62 is highly conserved among all oxyphototrophs, the C-terminal region (FNR-binding module) is only found in vascular plants. Phylogenetic analyses classify four Tic62-NAD(P)-related protein subfamilies in land plants, closely related to members from cyanobacteria and green sulphur bacteria. Although most of the Tic62-NAD(P)-related eukaryotic proteins are localized in the chloroplast, one subgroup consists of proteins without a predicted transit peptide. The N-terminal module of Tic62 contains the structurally conserved Rossman fold and probably belongs to the extended family of short-chain dehydrogenases-reductases. Key residues involved in NADP-binding and residues that may attach the protein to the inner envelope membrane of chloroplasts or to the Tic complex are proposed.

**Conclusion:**

The Tic62-NAD(P)-related proteins are of ancient origin since they are not only found in cyanobacteria but also in green sulphur bacteria. The FNR-binding module at the C-terminal region of the Tic62 proteins is probably a recent acquisition in vascular plants, with no sequence similarity to any other known motifs. The presence of the FNR-binding domain in vascular plants might be essential for the function of the protein as a Tic component and/or for its regulation.

## Background

Chloroplasts, together with mitochondria, are the major energy producers in all eukaryotic photosynthetic organisms. The endosymbiotic theory proposes a prokaryotic origin for plastids and mitochondria. During the endosymbiotic process a host cell engulfed distinct ancestral bacteria. Part of the genomes of these endosymbiotic bacteria have been kept and, as a result, plastids and mitochondria are the only organelles in the cell containing their own genome. However, while the chloroplast genome is composed of about 120 genes, its proteome is estimated to consist of about 3000 proteins [1]. The development of highly specific organellar transport mechanisms was thus the response to the necessity for re-importing the gene products and to guarantee an optimal communication between cells and organelles.

The general import pathway in chloroplasts involves the cooperation of two heteroligomeric complexes in the outer and inner envelope of chloroplasts, namely the Toc complex–composed of Toc159, Toc75, Toc64, Toc34 and Toc12 subunits–and the Tic complex–made up of Tic110, Tic62, Tic55, Tic40, Tic32, Tic22 and Tic20 subunits–respectively [2]. For a proper import activity, several chaperones in the cytosol (Hsp90 and cHsp70), intermembrane space (isHsp70) and stroma (ClpC and Cpn60) are functionally coordinated during different stages of the transport process [3-5].

Unlike the different protein transport systems in thylakoids, the protein import machinery in the outer/inner envelope membrane of chloroplasts does not show obvious homology to any bacterial secretion system [6]. This is hardly surprising since the bacterial systems were required for thylakoids and therefore a new transport machinery had to be developed in the host cell to maintain the specificity for chloroplast communication. However, sequence analyses indicated that certain components of the translocons in chloroplasts are of bacterial origin. Besides, there is a parallelism in the chaperone system required in some transport stages [6]. The translocation channel in the outer envelope membrane, Toc75, is related to outer membrane proteins involved in the transport or integration of proteins in Gram-negative bacteria [7,8]. Tic20, which is discussed to constitute part of the protein-conducting channel, shares sequence similarities to bacterial amino acid transporters [9]. Other subunits might have been recruited and adapted as they show homology to bacterial proteins not related to transport processes. Tic22, which is thought to mediate the interaction of the Toc and Tic complexes during import, has cyanobacterial counterparts with unknown function and is proposed to be localized in the thylakoid lumen [10]. Some cyanobacterial proteins contain cofactor-binding motifs similar to those found in Tic62, Tic55 and Tic32. Tic55 contains a Rieske iron-sulphur centre and a mononuclear iron-binding site [11], and Tic62 and Tic32 each have a NAD(P)-binding motif [12,13]. No prokaryotic counterparts have been detected by direct sequence comparison for the other subunits that compose the translocons, which may indicate that they have evolved from the proteome of the ancestral host to fulfil specific functions demanded after the development of plastids and to ensure the specificity of the transport process in the outer/inner envelope membranes of chloroplasts.

Genome-wide analyses had shown that some subunits of the translocons (Toc75, Toc159, Toc34, Tic20) are encoded by more than one gene in *Arabidopsis thaliana *[14,15]. Experimental data derived from analyses of the isoforms of the Toc complex revealed that the different members associate with structurally and developmentally distinct import complexes. Four homologues compose the Toc75 family in *Arabidopsis thaliana *(atToc75-I, atToc75-III, atToc75-IV and atToc75-V). The gene encoding the functional orthologue of Toc75 from *Pisum sativum*, atToc75-III, is essential for the viability of plants from the embryonic stage. This is not the case for atToc75-IV, which could play a role during growth in the dark. It seems that atToc75-I is in fact a pseudogene [16]. The function and relation with the Toc machinery of atToc75-V is still a matter of intensive study. In the case of Toc34 and Toc159 families, two (atToc33 and atToc34) and four (atToc159, atToc132, atToc120 and atToc90) isoforms are identified in the *Arabidopsis *genome, respectively. Whereas atToc33 associates preferably with atToc159, atToc34 does with atToc132/atToc120 and this association is likely related to the import of photosynthetic and non-photosynthetic precursors, respectively [17,18]. Four homologues are identified for Tic20 in *Arabidopsis *and only two of them contain a predicted transit peptide. However, the function and subcellular localization of the two Tic20 homologues with non-predicted transit peptide are still unknown [14].

In spite of the wealth of information about the Toc complex, less is known about phylogenetic relationships of the Tic complex subunits. Here we take a closer look at the structure, function and evolution of one component of the Tic complex, Tic62. The N-terminal module of Tic62 has a conserved NAD(P)-binding site and its C-terminal region was found to interact with ferredoxin-NAD(P)-oxido-reductase (FNR) [12]. Homology searches and phylogenetic analyses show that the N-terminal domain is highly conserved among all oxyphototrophs and green sulphur bacteria. However, the C-terminal region (FNR-binding domain) is only found in vascular plants. Phylogenetic analyses indicate that there are four groups of Tic62-NAD(P)-related proteins in land plants. The first group is orthologous to the reported Tic62 from pea [12]. The physiological roles of the Tic62-NAD(P)-related proteins in the cell remain to be shown.

## Results and discussion

Tic62, a protein of 62 kDa that is part of the Tic complex, has been proposed to function as a sensor protein whose possible role is to regulate protein import into chloroplasts by sensing and reacting to the redox state of the organelle. So far the only Tic62 protein studied is that from *Pisum sativum *[12]. This protein was found to have two functional modules: the N-terminus was shown to bind pyridine nucleotides and the C-terminal region interacts with FNR. The FNR-binding module consists of a repetitive, highly conserved KPPSSP motif. One or two transmembrane helices were proposed for the pea sequence and both the N- and the C-terminus seem to face the stroma [12].

**Figure 1:**
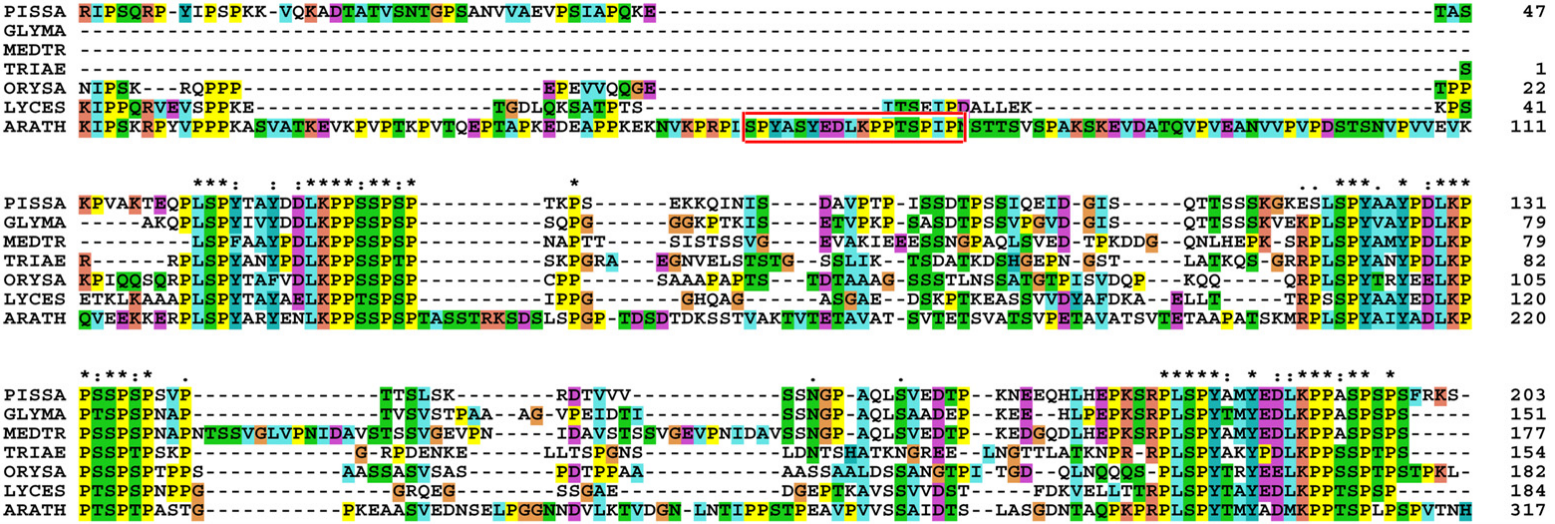
**Multiple sequence alignment of the C-terminal domain of the Tic62 protein family from vascular plants**. The multiple sequence alignment shows the FNR-binding domain in vascular plants (residues 387–534 in psTic62). Three repetitive motifs, S-P-Y-x (2)-Y-x-D/E-L-K-P (2)-S/T/A-S/T-P-S/T-P, involved in the binding of FNR [12] are highly conserved within the family. A fourth repetition found just in *Arabidopsis *sequence is marked in a box. The pea (PISSA), *Arabidopsis thaliana *(ARATH) and *Oryza sativa *(ORYSA) sequences were retrieved from GenBank. The sequences from tomato (LYCES), *Glycine max *(GLYMA), *Medicago truncatula *(MEDTR) and *Triticum aestivum *(TRIAE) were identified in dbEST and retrieved from plantGDB. The representation of the alignment is the standard from the ClustalX program [43].

Excluding the transit peptide, psTic62 consists of 470 residues. The blast search against the protein databases with psTic62 [Swiss-Prot:Q8SKU2] as a template resulted in several sequences from which just two correspond to the full-length form of the mature psTic62: one from *Arabidopsis thaliana *[GenBank:NP_188519] and another from *Oryza sativa *[GenBank:ABG65881]. All the other hits, which showed recognizable sequence similarity to the N-terminal NAD(P)-binding domain of Tic62, lack the C-terminal module (residues 387–534) responsible for the FNR binding and represent a short version of the Tic62 protein. A search of the FNR-binding motif over dbEST revealed its presence exclusively in vascular plant organisms (*e.g.*, *Lycopersicon esculentum*, *Medicago truncatula*, *Triticum aestivum*, *Glycine max*, *Lotus japonica*) (Figure 1).

**Figure 2:**
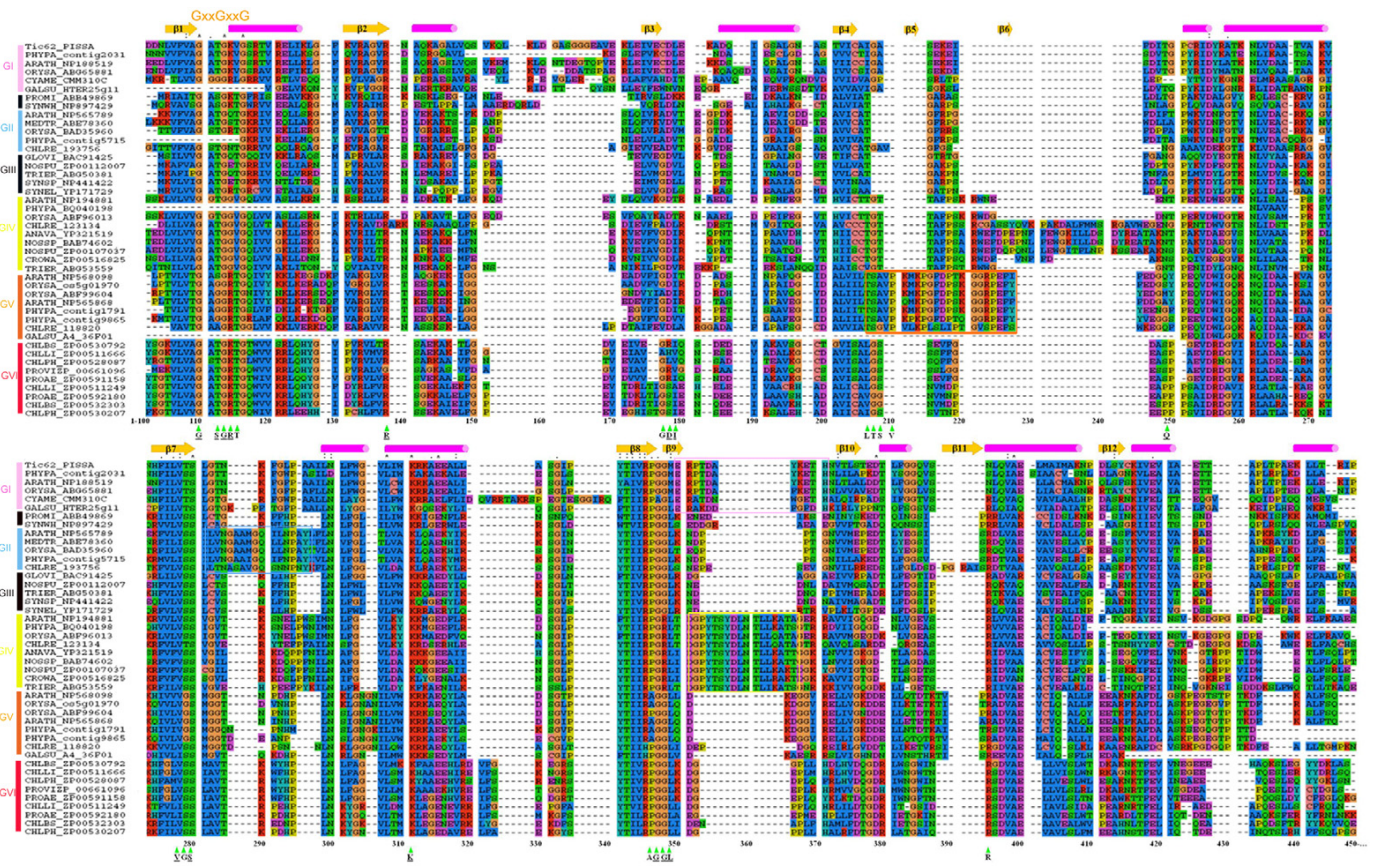
**Multiple sequence alignment of the N-terminal domain of the Tic62-NAD(P)-related protein family**. A multiple sequence alignment of the N-terminal domain (residues 87–334 in psTic62) of representative members of each of the six groups of Tic62-NAD(P)-related sequences was performed with ClustalX. Above the alignment the site of the GxxGxxG motif and the known secondary structure of the NP_568098 sequence from *Arabidopsis *[PDB:1XQ6] are displayed. α-helices and β-strands are represented by cylinders and arrows, respectively. The positions of the residues involved in the binding of NADP are marked with a triangle, and the identities of the residues from the crystal structure are indicated. The conserved residues between 1XQ6 and psTic62 are underlined. The sequence motifs that distinguish each group are shown in a box (see Results and Discussion). In the alignment the sequences are indicated with an abbreviation of the name of the organism followed by its identifying access code in the databases: ANAVA, *A. variabilis *ATCC 29413; ARATH, *A. thaliana*; CHLBS, *C. phaeobacteroides *BS1; CHLLI, *C. limicola *DSM 245; CHLPH, *C. phaeobacteroides *DSM 266; CHLRE, *C. reinhardtii*; CROWA, *C. watsonii *WH 8501; CYAME, *C. merolae*; GALSU, *G. sulphuraria*; GLOVI, *G. violaceus *PCC 7421; MEDTR, *M. truncatula*; NOSPU, *N. punctiforme *PCC 73102; NOSSP, *Nostoc sp. *PCC 7120; ORYSA, *O. sativa*; PHYPA, *P. patens*; PROAE, *P. aestuarii *DSM 271; PROMI, *P. marinus *str. MIT 9312; PROVI, *P. vibrioformis *DSM 265; SYNEL, *S. elongatus *PCC 6301; SYNSP, *Synechocystis sp. *PCC 6803; SYNWH, *Synechococcus sp*. WH 8102; TRIER, *T. erythraeum *IMS101. Note that the sequences from GALSU (GI and GIV), PHYPA (GII and GIII) and CHLRE (GIII and GIV) are incomplete sequences and lack part of the N- and C-terminal region. The representation of the alignment is the standard from the ClustalX program [43].

**Figure 3:**
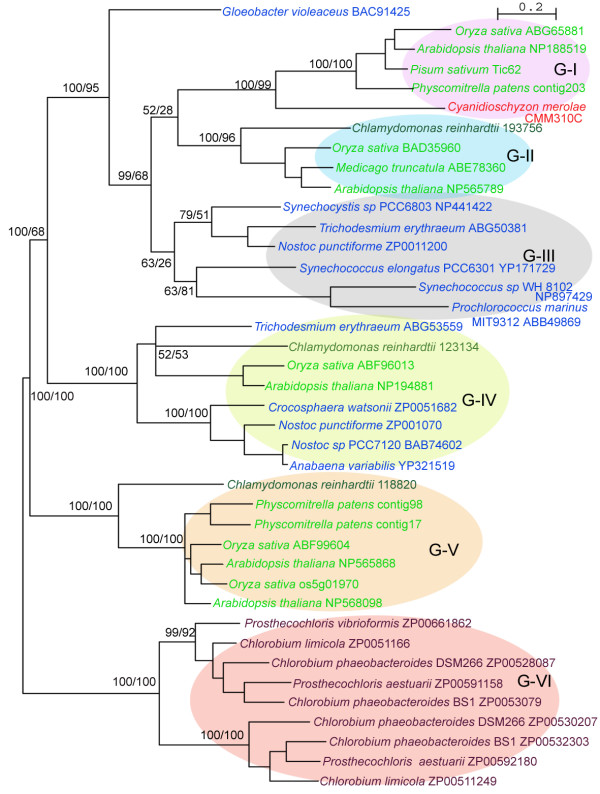
**Phylogram of representative members of the Tic62-NAD(P)-related family**. The optimal unrooted phylogenetic tree obtained by MrBayes is shown for representative members of the Tic62-NAD(P)-related family. The topology predicted with Bayesian and ML methods were not different from each other and four well-supported clusters and six groups are recognized. For display purposes, the green sulphur bacteria have been used as outgroup. The Bayesian posterior probability percentage (pP%) and the bootstrap values obtained by PhyML are shown in the nodes (Bayesian/ML). The organism's name is indicated followed by the accession number of the protein in the databases. Land plants, green algae, red algae, cyanobacteria and green-sulphur bacteria are coloured in light green, dark green, red, blue and brown, respectively. Branch lengths are proportional to evolutionary distances.

**Figure 4:**
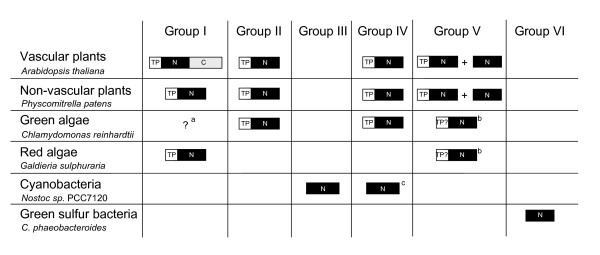
**Presence of Tic62-NAD(P)-related proteins in cyanobacteria, algae, land plants and green sulphur bacteria**. Tic62 is a bimodular protein (Nt and Ct modules for NAD(P) and FNR binding, respectively) with a transient transit peptide (TP) for importing into chloroplasts. The presence of the modules and/or the transit peptide is indicated for the Tic62-NAD(P)-related proteins. A question mark indicates that the complete genome is not available and: ^a ^the absence of the protein cannot be assured; ^b ^the existence of a transit peptide is not known. ^c^Proteins corresponding to group IV are only found in unicellular and filamentous diazotrophic cyanobacteria and not in others such as *Gloeobacter violaceus*, *Prochlorococcus marinus *or *Synechocystis sp. *PCC 6803 (see text). The accession numbers in the corresponding database (indicated in brackets) for the proteins found in this figure are the following: *Arabidopsis thaliana *(GenBank), NP_188519 (group I), NP_565789 (group II), NP_568098/NP_565868 (group IV, without/with transit peptide), NP_194881 (group V); *Physcomitrella patens *(PhyscoDB), contig 2031 (group I), contig 5715 (group II), contig1791/contig9865 (group IV, without/with transit peptide), BQ040198 (group V); *Chlamydomonas reinhardtii *(ChlamyDB), 193756 (group II), 118820 (group IV), 123134 (group V); *Galdieria sulphuraria *(The *Galdieria sulphuraria *Genome Project), hter25g11 (group I), A436F01 (group IV); *Nostoc sp. *PCC7120 (GenBank), ZP_00112007 (group III), BAB74602 (group IV); *Chlorobium phaeobacteroides *(GenBank), ZP_00528087 (group VI).

Interestingly, all the proteins homologous to the NAD(P)-binding part of Tic62 were from photosynthetic organisms (green plants, oxyphotobacteria, and green sulphur bacteria). A multiple sequence alignment of these proteins is shown in Figure 2. A phylogenetic tree was built based on the alignment (Figure 3). Both the multiple alignment and the phylogenetic tree indicate that the Tic62-NAD(P)-related protein family is made up of four well-supported clusters (support values of 100/95, 100/68, 100/100 and 100/100, Figure 3) that have been divided into six groups. These groups are schematically represented in Figure 4 and are described below. The four plant subfamilies are classified according to the GenBank accession number of the *Arabidopsis *protein found within each group (the locus_tag of the *Arabidopsis *gene is shown in parenthesis).

(i) Group I: NP_188519 (At3g18890). This subfamily contains the original Tic62 sequence from pea and makes up the Tic62 family, even though not all the members of this family have a molecular weight of 62 kDa and the association with the Tic machinery remains to be shown (see below). It is composed of proteins from chloroplast-containing organisms of land plants and red algae. So far no sequence of this subfamily was found in green algae (*Ostreococcus *or *Chlamydomonas*), in the diatom *Thalassiosira *or in any oxyphotobacteria. Because a final annotation of the green algae genomes is still in progress, a final confirmation of the absence of the protein of group I in green algae is pending. This group is characterized by the motif E-R-P/A-T-D-X-Ar-K/G-E-T-H (residues 350–371 in Figure 2), where Ar represents an aromatic residue. Surprisingly, only the sequences from vascular plants within this group show the full-length version of the Tic62 protein and contain the FNR-binding motif at the C-terminus. A minor distinction between Tic62 from *Arabidopsis *and the other full-length sequences is the number of four or three repetitive modules, respectively (Figure 1). Exhaustive searches for the FNR-interacting repeat in the *Physcomitrella patens *genome revealed no hits to these regions. 3'RACE PCRs of the detected *Physcomitrella *Tic62 gene were performed to determine its C-terminal sequence. It resulted exclusively in the short form of the gene, giving a stop codon in position 259. Additionally, immunodecoration with the pea Tic62 antibody, raised against the C-terminal part of the protein (residues 412–534), showed no signal in *Physcomitrella *chloroplasts (data not shown). Finally, an insertion of 6–15 residues (positions 148–168 in the alignment, Figure 2) is found in vascular plants and red algae. The search of this motif in the *Physcomitrella *genome resulted in no hits. The overall identity of the sequences composing this subfamily is 40%. All of them contain a transit peptide for targeting the protein to chloroplasts, and they might be localized at the inner envelope membrane as it was previously reported for the pea sequence [12], though the FNR-binding domain could modulate the subcellular localization of the protein by the interaction with FNR and/or other proteins.

(ii) Group II: NP_565789 (At2g34460). The members of this subfamily are homologous to the short version of the Tic62 protein from vascular plants. This subfamily is composed of proteins from the green algae *Chlamydomonas reinhardtii*, and both non-vascular and vascular plants (*Physcomitrella *and *Arabidopsis*, respectively). Surprisingly, there is no sign of the presence of members of this group in red algae genomes (*Galdieria*, *Porphyra *or *Cyanidioschyzon*). This group is closely related to group I and the phylogenetic tree is highly consistent in splitting up these two groups (Figure 3). The green plant sequences of this group are characterized by the motif L-V-N-G-A-A-p-G-Q-x(2)-N-P-A-Y, where p represents a polar residue (range 282–296 in Figure 2). The proteins from green plants contain an N-terminal extension, which is predicted to act as a transit peptide to target the proteins to chloroplasts. Recently, the *Arabidopsis *protein has been identified in a proteomic analysis of isolated plastoglobules [19].

(iii) Group III. This cyanobacterial subfamily is composed of proteins from a variety of organisms such as *Synechocystis *sp. PCC 6803, the small-genome cyanobacteria *Prochlorococcus marinus *(MIT9313, SS120 and MED4) or the heterocystous cyanobacteria *Nostoc *sp. This group comes together with groups I and II in a well-supported cluster (support values 100/95) and the phylogenetic trees were highly consistent in outgrouping the sequence from *Gloeobacter violaceus *(Figure 3), a cyanobacterial member of an early branching lineage [20]. Due to the annotation in the databases of the *Synechocystis *sequence from this family (NP_441422, sll1218) as ycf39 gene product, a connection between Tic62 and ycf39 was previously proposed [12]. However, it can be traced that the original ycf39 gene product is not related to sll1218 but to slr0399 in *Synechocystis *[GenBank:NP_441851] [12]. Both cyanobacterial proteins share 26% identity and 42% similarity. The ycf39 gene product (slr0399) was found to act as a chaperone for quinone binding [21]. This cyanobacterial protein is similar to the NP_195251*Arabidopsis *sequence that is not a Tic62-NAD(P)-related protein. Therefore, it can be argued that a connection between Tic62 and ycf39 may be an artefact originated by a non-reliable annotation in the protein database.

(iv) Group IV, NP_194881 (At4g31530). This group is made up of proteins from green plants and, interestingly, only unicellular and filamentous diazotrophic cyanobacteria. The members of this family also represent short versions of the Tic62 protein. So far no sequences from red algae were found. This group is characterized by the motif G-P-Y-T-S-Y-D-L-N-T-L-L-K/Q-A-T/K-A/S/T (range 353–377 in Figure 2). The land plant sequences contain a predicted chloroplast transit peptide and proteomics studies have localized the protein from *Arabidopsis *in chloroplasts [22]. The lack of homologous sequences in other cyanobacteria such as *Synechocystis *or *Prochlorococcus *may be in accordance with a previously reported work, which showed that *Nostoc *proteins have higher similarity to *Arabidopsis *nuclear-encoded proteins than proteins from *Prochlorococcus *or *Synechocystis *[23].

(v) Group V, NP_568098 (At5g02240). This family consists of proteins exclusively from eukaryotic phototrophs, which show homology to the short version of Tic62. The sequences of the land plant members of this group are characterized by the motif T-S-A-V-P-K-M-K-P-G-F-D-P-S/T-K-G-G-R-P-E-F-h, where h represents a hydrophobic residue (range 206–227 in Figure 2). Two different subgroups of sequences within this subfamily in land plants were identified, which are differentiated by the presence of a predicted transit peptide. This may suggest a dual localization in the cell for the members of this group. The first subgroup comprises land plant proteins without a predicted transit peptide. These proteins are [GenBank:NP_568098] (At5g02240), [GenBank:AAK73149] (Os03g60740) and [PhyscoDB:contig1791] from *Arabidopsis*, rice and *Physcomitrella*, respectively. The second subgroup is composed of sequences that contain a predicted transit peptide for chloroplasts: [GenBank:NP_565868] (At2g37660), [GenBank:ABF99604] (Os05g01970) and [PhyscoDB:contig9865] from *Arabidopsis*, rice and *Physcomitrella*, respectively. At2g37660 has been found in chloroplasts by proteomics analyses [22]. In spite of a possible difference in localization, the two subgroups are highly similar (*e.g.*, 79% identity between NP_568098 and NP_565868 in *Arabidopsis*) which suggests a similar function in the cell. Only incomplete sequences were found in the algae genomes analysed (*Chlamydomonas *and *Galdieria*) and, therefore, no further conclusions can be made for these organisms.

The structure of the NP_568098*Arabidopsis *protein bound to NADP has been recently solved at 1.8 Å resolution by X-ray crystallography [PDB:1XQ6]. The residues involved in the binding to the cofactor are marked with a triangle in the multiple sequence alignment (Figure 2).

(vi) Group VI. The last group to be mentioned corresponds to proteins of green sulphur bacteria (Figure 2 and Figure 4). Two subgroups are recognized, which likely originated from a gene duplication event (Figure 3). The similarity search using psTic62 as a template retrieved sequences from green sulphur bacteria with homology to the short version of Tic62. These are anoxygenic phototrophic bacteria that contain a type-I (Fe-S) reaction centre. A reverse blast search, using the green sulphur Tic62-related sequences, did not retrieve any sequences from oxyphotosynthetic organisms different from the groups mentioned above. Although very different organisms, the genome comparison between green sulphur bacteria and oxyphotosynthetic organisms showed that many components of photosynthesis and energy metabolism are highly similar. Green sulphur bacteria, cyanobacteria and eukaryotic phototrophs are the only organisms that synthesize chlorophyll *a *and also directly reduce pyridine nucleotides[24].

The presence of so many proteins in chloroplasts related to the NAD(P)-binding domain of Tic62 deserves a detailed study. The function of the N-terminal module seems important for the viability of the photosynthetic organisms since the gene has been conserved in all the genomes. All the proteins are predicted to bind pyridine nucleotides and are referred here as Tic62-NAD(P)-related family due to the similarity to the NAD(P)-binding domain of psTic62. The Tic62-NAD(P)-related family is of ancient origin, as proteins were not only found in ancient cyanobacteria (*Gloeobacter violaceus*) but also in green sulphur bacteria. This might propose that a Tic62-NAD(P)-related protein was already present in the ancestor who evolved to green sulphur bacteria and cyanobacteria. The presence of two genes in *Nostoc punctiforme *(groups III and IV) might suggest that a gene duplication event occurred prior to the evolution of cyanobacteria (Figure 3). Some cyanobacterial organisms could have lost one of the genes, which could explain its absence in *Gloeobacter*, *Prochlorococcus *and *Synechocystis *in group IV. Two highly supported groups (I and II) together with group III comprise a big cluster of sequences and groups I and II are possibly derived from group III which contains the majority of the cyanobacterial proteins. A four-cluster likelihood-mapping analysis (cluster a = group I+II, cluster b = group III, cluster c = group IV, cluster d = group V or cluster a = group I, cluster b = group II, cluster c = group IV, cluster d = group V) showed that branching order (a, b)–(c, d) was favoured in more than 90% of 10,000 puzzling, and demonstrated that group V is closely related to group IV. The presence of paralogues in land plants of group V could be due to a gene duplication event within the eukaryotic organism.

Most of the Tic62-NAD(P)-related proteins in higher plants are found in chloroplasts, but only the specific localization of psTic62 (group I) at the inner envelope membrane of chloroplasts and NP_565789 (At2g24460, group II) in plastoglobules have been shown experimentally [12,19]. It would be worth investigating the subcellular localization of the other members of the family and, especially, to analyse the possible dual localization of the proteins belonging to group V. The lack of a transit peptide had also been described in two homologues that compose the Tic20 family in *Arabidopsis *[14]. A possible localization outside plastids could be another example of a protein of cyanobacterial origin that has been redirected to a compartment different from plastids [23]. However, the targeting information to chloroplasts could be different from the canonical transit peptide [22,25,26] and a localization of such proteins in chloroplasts cannot be excluded. The presence of members of the family at the inner envelope membrane of chloroplasts, involved in the import process, and in plastoglobules, structures that act as a functional metabolic link between the inner envelope and thylakoid membranes, points to an important role of the protein family in metabolism.

The resolution of the structure of a protein is a major step in understanding the function. Since the similarity among sequences in the Tic62-NAD(P)-related family is sufficiently high, the knowledge of the structure of one member of Tic62-related family permits to draw general conclusions about the structure of other members. The crystallized NP_568098 protein shows the typical NADPH-Rossman fold. Figure 2 also represents the secondary structure of the crystallized NP_568098 protein. Clearly, most of the insertions and deletions of the proteins in this family correspond to loops in the crystal structure and most of the motifs related to α- and β-conformations are highly conserved. Therefore, the NADPH-Rossman fold is also expected for the core structure of all members of the Tic62-NAD(P)-related family, with differences mainly in the loop regions. The glycine-motif in the coenzyme-binding region is fully conserved in the whole family (GxxGxxG, range 111–116 in Figure 2) and it may be related to the extended short-chain dehydrogenase-reductase superfamily [27]. The highly conserved aspartic acid residue required for stabilization of the adenine-binding pocket is found in the loop between β3 and α3, except for group VI [28]. However, large differences are expected in the regions of the β5 and β6 strands. In the crystal structure, these two β-strands form an antiparallel β-sheet, which connect a long loop (Figure 2; see below). The differences in this region among the subfamilies could be correlated to the specific function of each subfamily. Since the protein was crystallized in the presence of NADP, the residues involved in the binding to the cofactor were identified (G11, S13, G14, R15, T16, R38, G55, D56, I57, L76, T77, S78, A79, V80, Q103, V131, G132, S133, K155, A174, G175, G176, L177, R205; for underlined residues see below). These residues are marked in Figure 2. From the multiple sequence alignment it can be concluded that many residues that bind to NADP are highly conserved within the family (9 out of 22). Specifically, the conservation of the residues (or their physicochemical properties) involved in the NADP binding is high in members of group I (14 out of 22). These residues are underlined above and they could represent the residues implicated in the NADP binding of Tic62. Mutagenesis studies are necessary to establish the role of these residues clearly.

**Figure 5:**
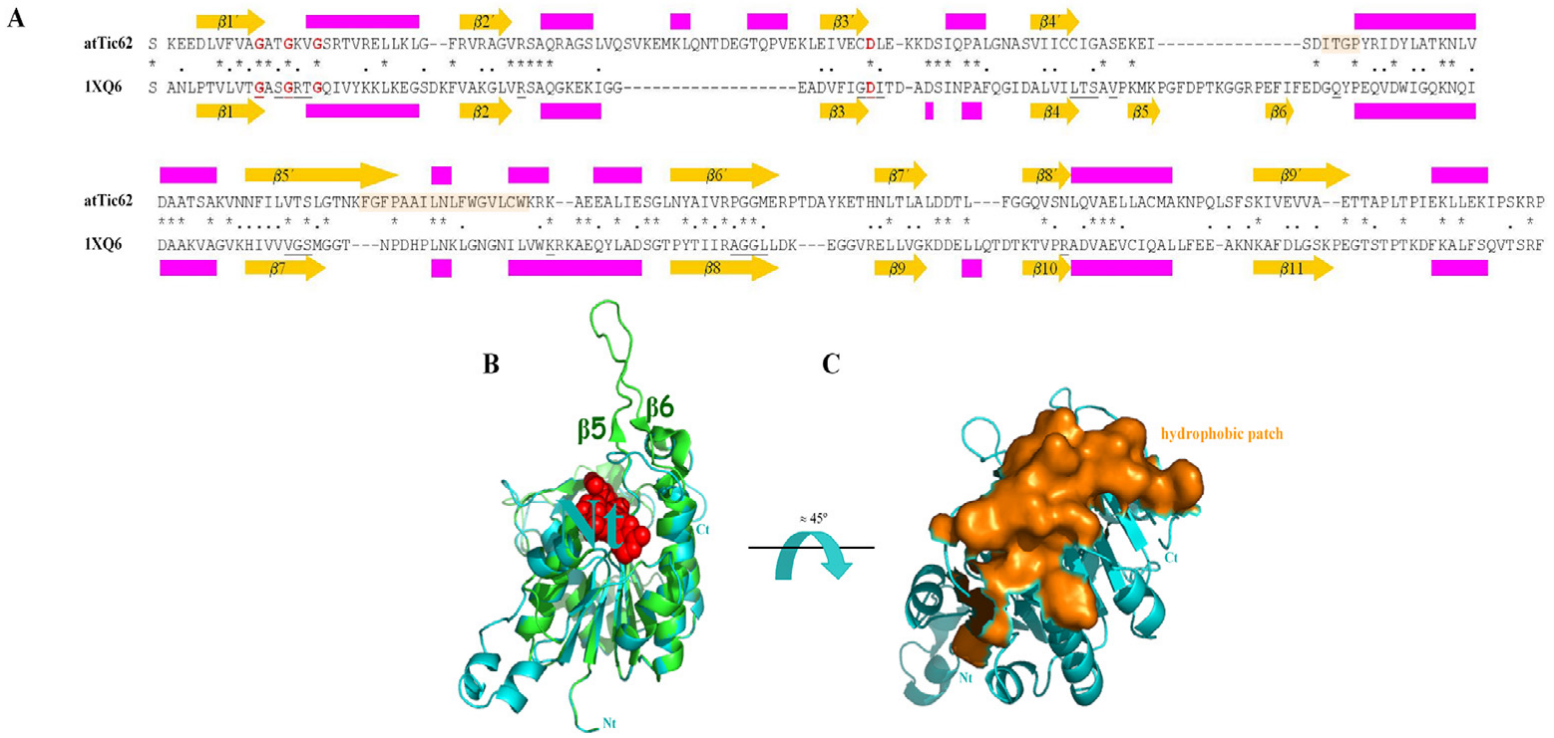
**3D structural model of the N-terminal domain of Tic62 from *Arabidopsis thaliana***. The model was built by homology modelling using the 1XQ6 structure as a template. (a) Sequence alignment of the N-terminal domain of the atTic62 (residues 78–331) protein and the template. The predicted and known secondary structure of atTic62 and the template (1XQ6) are shown. Purple represents α-helix and yellow denotes β-strand. Residues involve in NADP-binding are underlined. The conserved aspartic acid residue required for stabilization of the adenine-binding pocket is found at the end of β3. For residues shade in orange see below; (b) Proposed structural model for atTic62 (cyan) superimposed onto the template (green). NADP ligand is shown in red. Slight differences are expected among subfamilies (*e.g.*, absence of β5 and β6 in Tic62); (c) The hydrophobic region in Tic62 that might attach the protein to the membrane/Tic complex (residues 180–184 and 217–233 in atTic62) is shown in orange.

The mode of interaction of Tic62 with the membrane/Tic complex is unknown. Previous experiments showed that likely hydrophobic contacts mediate the binding to the membrane/Tic complex, as most of the protein remains within the membrane upon alkaline and urea treatments [12]. TMHMM [29] and PredictProtein [30] algorithms do not predict any transmembrane helices in group I. Moreover, protease digestion experiments showed that psTic62 is protected in inner envelope vesicles that, together with the hydrophilic profile of Tic62, suggest that the protein faces the stroma while attached to the membrane/Tic complex. Based on the identity (27% identity; 41% similarity) between atTic62 (NP_188519) and NP_568098 [PDB:1XQ6], a homology model procedure was followed to construct a model for the NADP-binding domain of the Tic62 protein (residues 78–331 in atTic62; see additional file 1: PDB coordinates for the atTic62 model). Figure 5a shows the sequence alignment of the N-terminal domain of the atTic62 protein and the template based on the multiple sequence alignment of the Tic62-NAD(P)-related family (Figure 2). The key residues involved in the pyridine ring binding are shown in red. The predicted secondary structure of atTic62 is compared with the known secondary structure of the template. As can be seen, most of the conformational elements are conserved in both sequences. Slight differences are the presence of β5 and β6 strands in the template (as mentioned above), and two smalls α-helices predicted between β2 and β3 strands in atTic62. A model was built based on this alignment and it was structurally evaluated with WHATCHECK. The corresponding values were good: Ramachandran plot, -2.215; backbone conformation, -3.761; chi-1/chi-2 rotamer normality, -1.150; bond lengths, 0.716; bond angles, 1.439. Only the values for the backbone conformation were poor, but this is probably due to gaps in the alignment and located in loop regions of the template (Figure 5a). In fact, the structural analysis obtained by the VERIFY3D program assigns positive values all over the structure, except in the regions LQNTDEGT and FPAAILNLFWGVLC in atTic62 (minimum value of -0.16) that support the previous proposal. The energetic parameter of the model was *E *= -4082.780 kJ·mol^-1^. In Figure 5b, a view of the proposed structural model for at Tic62 (blue) superimposed to the template (green) is despicted. The NADP ligand is shown in red and the residues involved in the binding are underlined in Figure 5a. It can be seen that the β5 and β6 elements connect a long loop that is missing in atTic62 (Figure 5a and 5b). Interestingly, a large number of hydrophobic residues is concentrated in this region in atTic62 (Figure 5c, marked in orange). The model presented here for atTic62 suggests that the hydrophobic region (residues 180–184 and 217–233 in atTic62 sequence in Figure 5c, which correspond to residues 247–251 and 291–310 in the alignment shown in Figure 2) might be responsible for attaching the protein to the inner envelope membrane of chloroplasts or to the Tic complex, and this region would establish differences in the localisation within cells between the two groups of proteins (template and model). By this way, Tic62 would be attached to the membrane, without spanning it, exposing the two functional modules to the stromal side. The hydrophilic profile and the large number of conserved proline residues at the C-terminal domain make it a better candidate for protein-protein interactions rather than for insertion into the membrane [31]. These interactions might also contribute to the binding of Tic62 to the membrane/Tic complex.

Focusing on group I, one of the questions to be answered is whether or not all members of this group are Tic components. Although they might share a common dehydrogenase activity at the N-terminus, the origin of the FNR-binding module at the C-terminus in vascular plants remains unknown and different functions might be expected among the different organisms. No similar sequences to the C-terminus of psTic62 were found in the databases with significant homology, which could indicate that either the FNR-binding module was lost during evolution and only kept in vascular plants, or (more probably) the FNR-binding module was recently acquired by vascular plants. The high similarity of the NADP-binding domain of the *Physcomitrella *sequence in group I with psTic62 (68% identity) suggests that the short version of Tic62 in *Physcomitrella*, together with Tic110/Tic55/Tic40/Tic32/Tic22/Tic20 [32], might be a constituent of the Tic complex. The same might be true for *Cyanidioschyzon merolae*. On the other hand, it cannot be excluded that the concurrence of both N-t and C-t domains, or even the FNR-binding domain alone, were compulsory to settle a protein as a Tic component. Further studies are necessary to establish the mode of interaction of Tic62 with the Tic complex in vascular plants and to elucidate the localization and function of members of group I in non-vascular plants.

Still the question remains of the presence and function of FNR at the inner envelope membrane of chloroplasts. In chloroplasts, this protein is found either soluble in the stroma in a non-functional state or attached to the thylakoids, where the protein is involved in the last stage of the electron transport process in photosynthesis. FNR is a ubiquitous flavoenzyme whose function is not exclusively confined to photosynthesis [33] and, recently, the protein has also been found to be localized at the inner envelope membrane of chloroplasts [12]. When attached to the thylakoids, a reductase-binding protein (BP) mediates the binding to the membrane [34]. In line with this, one possibility could be that FNR is attached to the inner envelope membrane via the FNR-binding motif of Tic62. This interaction in vascular plants could be affected by the activity of Tic62 that could specifically regulate the–yet unknown–functional state of FNR in the inner envelope membrane of chloroplasts. The opposite effect cannot be excluded, and the binding of FNR could regulate the activity of Tic62, and therefore the transport machinery. This regulation upon binding could depend on the redox state of chloroplasts and might involve NADP(H)/NAD(H) or a low potential electron donor and another substrate not yet identified [33]. On the other hand, a possible electron transfer process between FNR and Tic62 cannot be excluded although the capacity of Tic62 as electron acceptor/donor has not yet been proven. It is likely that the FNR-binding domain is important for some kind of metabolic regulation just in vascular plants, which needs further studies.

## Conclusion

The reported results show that the N-terminal module of Tic62 (NAD(P)-binding domain) is highly conserved among all oxyphototrophs. The Tic62-NAD(P)-related sequences are of ancient origin, since the protein was not only found in cyanobacteria but also in green-sulfur bacteria. This protein family would belong to the extended family of short-chain dehydrogenases-reductases and likely contains the structurally conserved Rossman fold. On the other hand, the C-terminal module in Tic62 (FNR-binding domain) is only found in vascular plants. This domain is enriched in proline amino acids and it would be important for protein-protein interactions that might regulate the function of Tic62 protein. Tic62 proteins in vascular plants would be attached to the inner envelope membrane of chloroplasts, without spanning it, exposing both C-terminal and N-terminal domains to the stroma. Further studies are necessary to establish the mode of interaction of Tic62 with the Tic complex in vascular plants and to elucidate the localization and function of related members in non-vascular plants

## Methods

A sequence homology search (tblastn/blastp) was performed using the Tic62 protein sequence from pea (psTic62) as a template (e-value < 10^-9^). The following biological databases were considered: the non-redundant GenBank database (nr) [35]; the public available *Physcomitrella patens *EST database, PhyscoDB [36]; the genomic database containing the so far sequenced *Physcomitrella patens *genome (access due to collaboration with Prof. Ralf Reski, University of Freiburg); the annotated genome of the red alga *Cyanidioschyzon merolae *[37]; the annotated genome of the green alga *Chlamydomonas reinhardtii*, ChlamyDB [38]; the genome database for plants, plantGDB [39]. Also the following databases were considered: the red algae *Porphyra yezoensis *[40] and *Galdieria sulphuraria *[41] databases; the chlorophyta *Ostreococcus lucimarinus *database [42]; the EST GenBank database (dbEST) [35]. All the retrieved sequences were aligned with the ClustalX program [43], visually inspected and manually corrected. The prediction of the subcellular localization of the proteins was performed with TargetP [44], ChloroP [45] and Predotar [46] programmes.

ProtTest v1.3 [47] was used to estimate the best model of amino acid evolution for phylogeny. The WAG+I+Γ model was chosen using either AIC or BIC as statistical frameworks. Phylogenetic trees were generated on the basis of the maximum-likelihood (ML) and Bayesian analysis using PhyML v2.4.4 [48] and MrBayes v3.1.2 [49] programmes. For ML analysis, four Gamma-distributed sites were considered, and the parameters were estimated from the data. Non-parametric bootstrap values were calculated for ML analyses (100 replicates) to assess the significance of the resulting tree. Bayesian analysis was performed under the same model. Four chains were run for one million generations with sampling every 100 generations. Bayesian posterior probabilities were calculated from the majority rule consensus of the tree sampled after the initial burn-in period corresponding to 2,500 generations. Four-cluster likelihood-mapping [50] implemented in Tree-puzzle v5.2 [51] was performed with 10,000 randomly chosen quartets.

A 3D model for all non-hydrogen atoms was obtained for the N-terminal domain of the mature Tic62 from *Arabidopsis thaliana *(atTic62; NP_188519) by homology modelling using the known 3D structure of NP_568098*Arabidopsis *protein [PDB:1XQ6] as a template. The model was built using the SWISS-MODEL automated modelling server [52] and it was evaluated using WHATCHECK [53], PROMODII [54] and VERIFY3D [55]. The secondary structure prediction of atTic62 was performed using PSIPRED server [56].

## Authors' contributions

MB carried out the acquisition, analysis and interpretation of the data, and drafted the manuscript. AS performed the RACE-PCR and immunodecoration experiments in *Physcomitrella patens*. AS and BB participated in the sequence analyses and contributed to each draft of the manuscript. JS directed the study and content of the manuscript. All authors read and approved the final manuscript.

## Supplementary Material

Additional File 1 PDB coordinates for the atTic62 model. Model of atTic62 built by homology modelling using 1XQ6 structure as a template. Click here for file

## References

[B1] van Wijk KJ (2004). Plastid proteomics. Plant Physiology and Biochemistry.

[B2] Jarvis P, Robinson C (2004). Mechanisms of protein import and routing in chloroplasts. Current Biology.

[B3] Qbadou S, Becker T, Mirus O, Tews I, Soll J, Schleiff E (2006). The molecular chaperone Hsp90 delivers precursor proteins to the chloroplast import receptor Toc64. Embo Journal.

[B4] Soll J, Schleiff E (2004). Protein import into chloroplasts. Nature Reviews Molecular Cell Biology.

[B5] Becker T, Hritz J, Vogel M, Caliebe A, Bukau B, Soll J, Schleiff E (2004). Toc12, a novel subunit of the intermembrane space preprotein translocon of chloroplasts. Molecular Biology of the Cell.

[B6] Wickner W, Schekman R (2005). Protein translocation across biological membranes. Science.

[B7] Reumann S, Davila-Aponte J, Keegstra K (1999). The evolutionary origin of the protein-translocating channel of chloroplastic envelope membranes: Identification of a cyanobacterial homolog. Proceedings of the National Academy of Sciences of the United States of America.

[B8] Bolter B, Soll J, Schulz A, Hinnah S, Wagner R (1998). Origin of a chloroplast protein importer. Proceedings of the National Academy of Sciences of the United States of America.

[B9] Reumann S, Keegstra K (1999). The endosymbiotic origin of the protein import machinery of chloroplastic envelope membranes. Trends in Plant Science.

[B10] Fulda S, Norling B, Schoor A, Hagemann M (2002). The slr0924 protein of Synechocystis sp strain PCC 6803 resembles a subunit of the chloroplast protein import complex and is mainly localized in the thylakoid lumen. Plant Molecular Biology.

[B11] Caliebe A, Grimm R, Kaiser G, Lubeck J, Soll J, Heins L (1997). The chloroplastic protein import machinery contains a Rieske-type iron-sulfur cluster and a mononuclear iron-binding protein. Embo Journal.

[B12] Kuchler M, Decker S, Hormann F, Soll J, Heins L (2002). Protein import into chloroplasts involves redox-regulated proteins. Embo Journal.

[B13] Hormann F, Kuchler M, Sveshnikov D, Oppermann U, Li Y, Soll J (2004). Tic32, an essential component in chloroplast biogenesis. Journal of Biological Chemistry.

[B14] Reumann S, Inoue K, Keegstra K (2005). Evolution of the general protein import pathway of plastids (Review). Molecular Membrane Biology.

[B15] Smith MD (2006). Protein import into chloroplasts: an ever-evolving story. Canadian Journal of Botany-Revue Canadienne De Botanique.

[B16] Baldwin A, Wardle A, Patel R, Dudley P, Park SK, Twell D, Inoue K, Jarvis P (2005). A molecular-genetic study of the Arabidopsis Toc75 gene family. Plant Physiology.

[B17] Ivanova Y, Smith MD, Chen KH, Schnell DJ (2004). Members of the Toc159 import receptor family represent distinct pathways for protein targeting to plastids. Molecular Biology of the Cell.

[B18] Jarvis P, Chen LJ, Li HM, Pete CA, Fankhauser C, Chory J (1998). An Arabidopsis mutant defective in the plastid general protein import apparatus. Science.

[B19] Ytterberg AJ, Peltier JB, van Wijk KJ (2006). Protein profiling of plastoglobules in chloroplasts and chromoplasts. A surprising site for differential accumulation of metabolic enzymes. Plant Physiology.

[B20] Honda D, Yokota A, Sugiyama J (1999). Detection of seven major evolutionary lineages in cyanobacteria based on the 16S rRNA gene sequence analysis with new sequences of five marine Synechococcus strains. Journal of Molecular Evolution.

[B21] Ermakova-Gerdes S, Vermaas W (1999). Inactivation of the open reading frame slr0399 in Synechocystis sp PCC 6803 functionally complements mutations near the Q(A) niche of photosystem II - A possible role of slr0399 as a chaperone for quinone binding. Journal of Biological Chemistry.

[B22] Kleffmann T, Russenberger D, von Zychlinski A, Christopher W, Sjolander K, Gruissem W, Baginsky S (2004). The Arabidopsis thaliana chloroplast proteome reveals pathway abundance and novel protein functions. Current Biology.

[B23] Martin W, Rujan T, Richly E, Hansen A, Cornelsen S, Lins T, Leister D, Stoebe B, Hasegawa M, Penny D (2002). Evolutionary analysis of Arabidopsis, cyanobacterial, and chloroplast genomes reveals plastid phylogeny and thousands of cyanobacterial genes in the nucleus. Proceedings of the National Academy of Sciences of the United States of America.

[B24] Frigaard NU, Chew AGM, Li H, Maresca JA, Bryant DA (2003). Chlorobium tepidum: insights into the structure, physiology, and metabolism of a green sulfur bacterium derived from the complete genome sequence. Photosynthesis Research.

[B25] Nada A, Soll J (2004). Inner envelope protein 32 is imported into chloroplasts by a novel pathway. Journal of Cell Science.

[B26] Miras S, Salvi D, Ferro M, Grunwald D, Garin J, Joyard J, Rolland N (2002). Non-canonical transit peptide for import into the chloroplast. Journal of Biological Chemistry.

[B27] Kallberg Y, Oppermann U, Jornvall H, Persson B (2002). Short-chain dehydrogenases/reductases (SDRs) - Coenzyme-based functional assignments in completed genomes. European Journal of Biochemistry.

[B28] Filling C, Berndt KD, Benach J, Knapp S, Prozorovski T, Nordling E, Ladenstein R, Jornvall H, Oppermann U (2002). Critical residues for structure and catalysis in short-chain dehydrogenases/reductases. Journal of Biological Chemistry.

[B29] Krogh A, Larsson B, von Heijne G, Sonnhammer ELL (2001). Predicting transmembrane protein topology with a hidden Markov model: Application to complete genomes. Journal of Molecular Biology.

[B30] Rost B, Yachdav G, Liu JF (2004). The PredictProtein server. Nucleic Acids Research.

[B31] Williamson MP (1994). The Structure and Function of Proline-Rich Regions in Proteins. Biochemical Journal.

[B32] Hofmann NR, Theg SM (2003). Physcomitrella patens as a model for the study of chloroplast protein transport: conserved machineries between vascular and non-vascular plants. Plant Molecular Biology.

[B33] Carrillo N, Ceccarelli EA (2003). Open questions in ferredoxin-NADP(+) reductase catalytic mechanism. European Journal of Biochemistry.

[B34] Vallejos RH, Ceccarelli E, Chan R (1984). Evidence for the existence of a thylakoid intrinsic protein that binds ferredoxin-NADP+ Oxidoreductase. Journal of Biological Chemistry.

[B35] Benson DA, Boguski MS, Lipman DJ, Ostell J, Ouellette BFF, Rapp BA, Wheeler DL (1999). GenBank. Nucleic Acids Research.

[B36] Physcomitrella patens EST genome. http://moss.nibb.ac.jp/.

[B37] Cyanidioschyzon merolae genome database. http://merolae.biol.s.u-tokyo.ac.jp/.

[B38] Chlamydomonas reinhardtii annotated genome database.

[B39] Plant genomes database. http://www.plantgdb.org/.

[B40] Porphyra yezoensis genome database. http://www.kazusa.or.jp/en/plant/porphyra/EST/index.html.

[B41] Galdieria sulphuraria genome database. http://genomics.msu.edu/cgi-bin/galdieria/blast.cgi.

[B42] Ostreococcus lucimarinus genome database. http://genome.jgi-psf.org/Ost9901_3/Ost9901_3.home.html.

[B43] Thompson JD, Gibson TJ, Plewniak F, Jeanmougin F, Higgins DG (1997). The CLUSTAL_X windows interface: flexible strategies for multiple sequence alignment aided by quality analysis tools. Nucleic Acids Research.

[B44] Emanuelsson O, Nielsen H, Brunak S, von Heijne G (2000). Predicting subcellular localization of proteins based on their N-terminal amino acid sequence. Journal of Molecular Biology.

[B45] Emanuelsson O, Nielsen H, Von Heijne G (1999). ChloroP, a neural network-based method for predicting chloroplast transit peptides and their cleavage sites. Protein Science.

[B46] Small I, Peeters N, Legeai F, Lurin C (2004). Predotar: A tool for rapidly screening proteomes for N-terminal targeting sequences. Proteomics.

[B47] Abascal F, Zardona R, Posada D (2005). ProtTest: Selection of best-fit models of protein evolution. Bioinformatics.

[B48] Guindon S, Gascuel O (2003). A simple, fast, and accurate algorithm to estimate large phylogenies by maximum likelihood. Systematic Biology.

[B49] Ronquist F, Huelsenbeck JP (2003). MrBayes3: Bayesian phylogenetic inference under mixed models. Bioinformatics.

[B50] Strimmer K, vonHaeseler A (1997). Likelihood-mapping: A simple method to visualize phylogenetic content of a sequence alignment. Proceedings of the National Academy of Sciences of the United States of America.

[B51] Schmidt HA, Strimmer K, Vingron M, von Haeseler A (2002). TREE-PUZZLE: maximum likelihood phylogenetic analysis using quartets and parallel computing. Bioinformatics.

[B52] Guex N, Diemand A, Peitsch MC (1999). Protein modelling for all. Trends in Biochemical Sciences.

[B53] Vriend G (1990). What If - a molecular modeling and drug design program. Journal of Molecular Graphics.

[B54] Peitsch MC (1996). ProMod and Swiss-model: Internet-based tools for automated comparative protein modelling. Biochemical Society Transactions.

[B55] Luthy R, Bowie JU, Eisenberg D (1992). Assessment of protein models with 3-Dimensional profiles. Nature.

[B56] McGuffin LJ, Bryson K, Jones DT (2000). The PSIPRED protein structure prediction server. Bioinformatics.

